# 4-Bromo-2-(dieth­oxy­meth­yl)phenyl benzoate

**DOI:** 10.1107/S1600536813006351

**Published:** 2013-03-16

**Authors:** P. Sharmila, C. Suresh Kumar, S. Maheshwaran, S. Narasimhan, S. Aravindhan

**Affiliations:** a Department of Physics, Presidency College, Chennai 600 005, India; b Asthagiri Herbal Research Foundation, Perungudi, Chennai 600 096, India

## Abstract

In the title compound, C_18_H_19_BrO_4_, the aromatic rings enclose a dihedral angle of 81.9 (7)°. There are no short directional contacts in the crystal structure.

## Related literature

For the biological activity of ester derivatives, see: Bi *et al.* (2012 ▶); Bartzatt *et al.* (2004 ▶); Anadu *et al.* (2006 ▶).
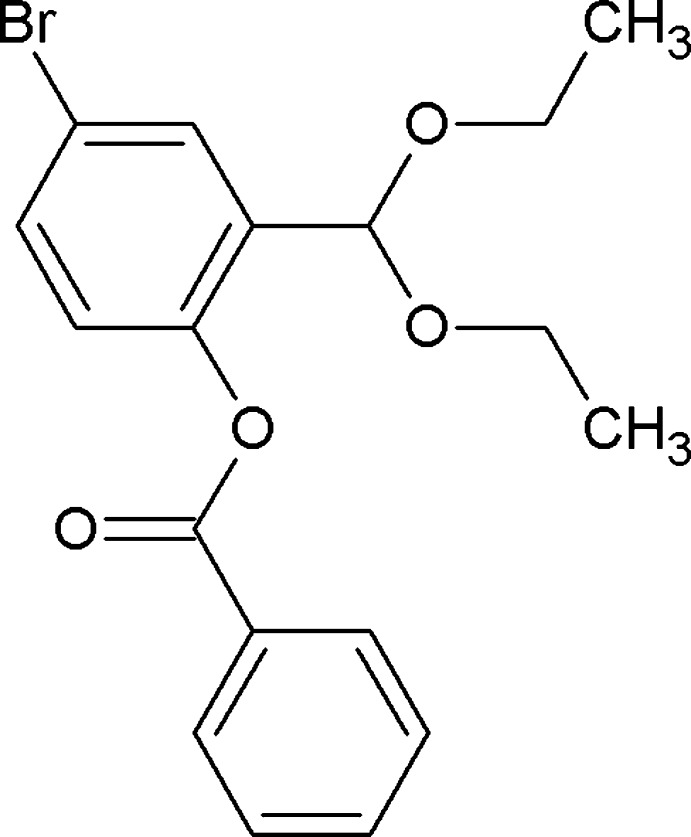


## Experimental

### 

#### Crystal data

C_18_H_19_BrO_4_
*M* *_r_* = 379.24
Triclinic, 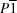

*a* = 8.2662 (8) Å
*b* = 9.6378 (10) Å
*c* = 11.6224 (13) Å
α = 99.927 (5)°
β = 93.700 (5)°
γ = 101.178 (5)°
*V* = 890.16 (16) Å^3^
*Z* = 2
Mo *K*α radiation
μ = 2.33 mm^−1^
*T* = 293 K
0.35 × 0.30 × 0.25 mm

#### Data collection

Bruker Kappa APEXII CCD diffractometer
Absorption correction: multi-scan (*SADABS*; Bruker 2004 ▶) *T*_min_ = 0.497, *T*_max_ = 0.594
16152 measured reflections
4493 independent reflections
3037 reflections with *I* > 2σ(*I*)
*R*_int_ = 0.035

#### Refinement

*R*[*F* ^2^ > 2σ(*F* ^2^)] = 0.034
*wR*(*F* ^2^) = 0.085
*S* = 1.01
4493 reflections
209 parameters
H-atom parameters constrained
Δρ_max_ = 0.46 e Å^−3^
Δρ_min_ = −0.42 e Å^−3^

### 

Data collection: *APEX2* (Bruker, 2004 ▶); cell refinement: *APEX2* and *SAINT* (Bruker, 2004 ▶); data reduction: *SAINT* and *XPREP* (Bruker, 2004 ▶); program(s) used to solve structure: *SHELXS97* (Sheldrick, 2008 ▶); program(s) used to refine structure: *SHELXL97* (Sheldrick, 2008 ▶); molecular graphics: *ORTEP-3 for Windows* (Farrugia, 2012 ▶); software used to prepare material for publication: *PLATON* (Spek, 2009 ▶).

## Supplementary Material

Crystal structure: contains datablock(s) I, global. DOI: 10.1107/S1600536813006351/bt6892sup1.cif

Structure factors: contains datablock(s) I. DOI: 10.1107/S1600536813006351/bt6892Isup2.hkl

Supplementary material file. DOI: 10.1107/S1600536813006351/bt6892Isup3.cml

Additional supplementary materials:  crystallographic information; 3D view; checkCIF report

## Figures and Tables

**Fig. 1.**
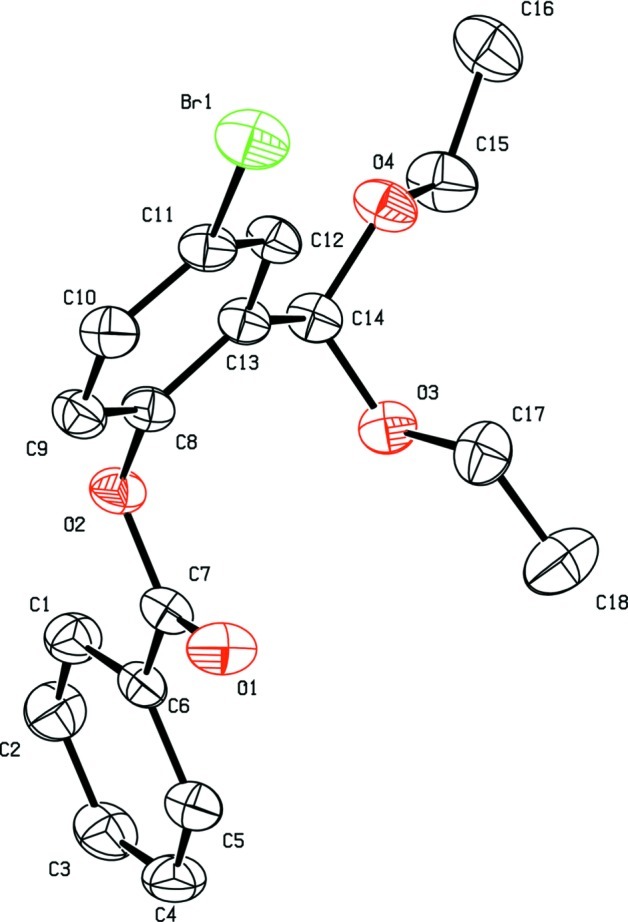
The molecular structure of the title compound, with the atom numbering scheme. Displacement ellipsoids are drawn at the 30% probability level. H atoms have been omitted for clarity.

**Fig. 2.**
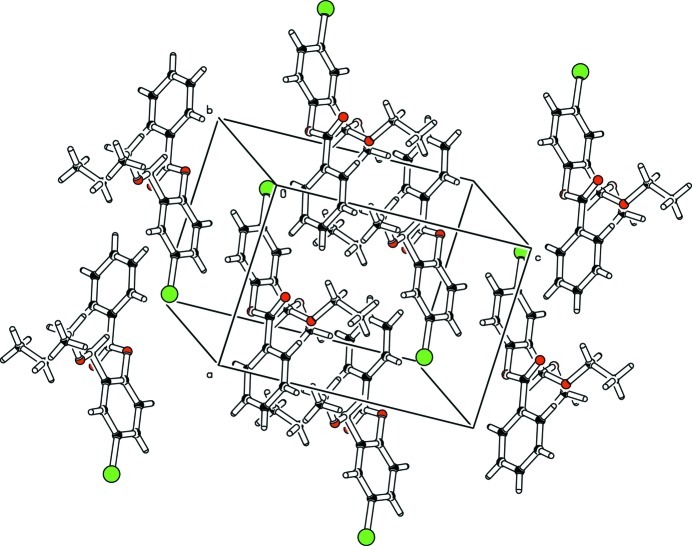
Crystal packing of the title compound viewed along the b axis. For the sake of clarity, H atoms have been omitted.

## supplementary crystallographic information

### Comment

Ester derivatives of many compounds exhibit a variety of pharmacological properties, for example anticancer, antitumor and antimicrobial activities (Anadu *et al.*, 2006; Bi *et al.*, 2012; Bartzatt *et al.*, 2004). In view of their importance, the title compound was synthesized and we report herein on its crystal structure.

In the title molecule (Fig. 1) the two aromatic rings enclose a dihedral angle of 98.1 (7)°. The molecular conformation is stabilized by C-H···O contacts. The crystals packing, on the other hand, shows no short contacts.

A packing diagram of the molecule is shown in Fig. 2.

### Experimental

A solution of 4-Bromo-2-diethoxymethyl-phenol (0.03 mol) in chloroform (100 ml) was cooled and Benzoyl chlorides (0.03 mol) was added dropwise followed by addition of triethyl amine (0.03 mol). Then, the reaction was stirred at room temperature for 3 h. The reaction mixture was quenched with water and the chloroform layer was separated. The combined chloroform layer was washed with 5% NaOH solution followed by water wash and dried with anhydrous sodium sulfate, concentrated under reduced pressure. The obtained solid was crystallized in a mixture of Methanol:Chloroform.

### Refinement

All the H atoms were positioned geometrically, with C–H = 0.93–0.97 Å and constrained to ride on their parent atom, with *U*_iso_(H) =1.5U_eq_(C) for methyl H atoms and 1.2U_eq_(C) for other H atoms.

### Crystal data

**Table:**

C_18_H_19_BrO_4_	*Z* = 2
*M**_r_* = 379.24	*F*(000) = 388
Triclinic, *P*1	*D*_x_ = 1.415 Mg m^−^^3^
Hall symbol: -P 1	Mo *K*α radiation, λ = 0.71073 Å
*a* = 8.2662 (8) Å	Cell parameters from 8834 reflections
*b* = 9.6378 (10) Å	θ = 2.1–31.2°
*c* = 11.6224 (13) Å	µ = 2.33 mm^−^^1^
α = 99.927 (5)°	*T* = 293 K
β = 93.700 (5)°	Block, colourless
γ = 101.178 (5)°	0.35 × 0.30 × 0.25 mm
*V* = 890.16 (16) Å^3^	

### Data collection

**Table:**

Bruker Kappa APEXII CCD diffractometer	4493 independent reflections
Radiation source: fine-focus sealed tube	3037 reflections with *I* > 2σ(*I*)
Graphite monochromator	*R*_int_ = 0.035
ω and φ scan	θ_max_ = 28.6°, θ_min_ = 1.8°
Absorption correction: multi-scan (*SADABS*; Bruker 2004)	*h* = −10→11
*T*_min_ = 0.497, *T*_max_ = 0.594	*k* = −12→12
16152 measured reflections	*l* = −14→15

### Refinement

**Table:**

Refinement on *F*^2^	Secondary atom site location: difference Fourier map
Least-squares matrix: full	Hydrogen site location: inferred from neighbouring sites
*R*[*F*^2^ > 2σ(*F*^2^)] = 0.034	H-atom parameters constrained
*wR*(*F*^2^) = 0.085	*w* = 1/[σ^2^(*F*_o_^2^) + (0.0325*P*)^2^ + 0.2669*P*] where *P* = (*F*_o_^2^ + 2*F*_c_^2^)/3
*S* = 1.01	(Δ/σ)_max_ = 0.001
4493 reflections	Δρ_max_ = 0.46 e Å^−^^3^
209 parameters	Δρ_min_ = −0.42 e Å^−^^3^
0 restraints	Extinction correction: *SHELXL97* (Sheldrick, 2008), Fc^*^=kFc[1+0.001xFc^2^λ^3^/sin(2θ)]^-1/4^
Primary atom site location: structure-invariant direct methods	Extinction coefficient: 0.0293 (17)

### Special details

**Table:**

Geometry. All e.s.d.'s (except the e.s.d. in the dihedral angle between two l.s. planes) are estimated using the full covariance matrix. The cell e.s.d.'s are taken into account individually in the estimation of e.s.d.'s in distances, angles and torsion angles; correlations between e.s.d.'s in cell parameters are only used when they are defined by crystal symmetry. An approximate (isotropic) treatment of cell e.s.d.'s is used for estimating e.s.d.'s involving l.s. planes.
Refinement. Refinement of *F*^2^ against ALL reflections. The weighted *R*-factor *wR* and goodness of fit *S* are based on *F*^2^, conventional *R*-factors *R* are based on *F*, with *F* set to zero for negative *F*^2^. The threshold expression of *F*^2^ > σ(*F*^2^) is used only for calculating *R*-factors(gt) *etc*. and is not relevant to the choice of reflections for refinement. *R*-factors based on *F*^2^ are statistically about twice as large as those based on *F*, and *R*- factors based on ALL data will be even larger.

### Fractional atomic coordinates and isotropic or equivalent isotropic displacement parameters (Å^2^)

**Table:**

	*x*	*y*	*z*	*U*_iso_*/*U*_eq_	
C1	1.0177 (3)	0.1162 (2)	0.14518 (19)	0.0568 (5)	
H1	1.0276	0.2024	0.1179	0.068*	
C2	1.1551 (3)	0.0569 (3)	0.1588 (2)	0.0668 (6)	
H2	1.2575	0.1035	0.1415	0.080*	
C3	1.1409 (3)	−0.0706 (3)	0.1979 (2)	0.0686 (6)	
H3	1.2335	−0.1108	0.2067	0.082*	
C4	0.9906 (3)	−0.1388 (2)	0.2238 (2)	0.0657 (6)	
H4	0.9814	−0.2257	0.2500	0.079*	
C5	0.8522 (3)	−0.0800 (2)	0.21151 (19)	0.0560 (5)	
H5	0.7504	−0.1266	0.2299	0.067*	
C6	0.8660 (2)	0.0487 (2)	0.17159 (16)	0.0446 (4)	
C7	0.7144 (3)	0.1076 (2)	0.16072 (17)	0.0476 (5)	
C8	0.6138 (2)	0.3097 (2)	0.12700 (17)	0.0454 (4)	
C9	0.4947 (3)	0.2747 (2)	0.03277 (18)	0.0530 (5)	
H9	0.5006	0.2030	−0.0306	0.064*	
C10	0.3663 (3)	0.3469 (2)	0.03332 (19)	0.0553 (5)	
H10	0.2848	0.3249	−0.0298	0.066*	
C11	0.3603 (2)	0.4517 (2)	0.12799 (18)	0.0495 (5)	
C12	0.4820 (2)	0.4902 (2)	0.22060 (17)	0.0477 (5)	
H12	0.4769	0.5639	0.2827	0.057*	
C13	0.6130 (2)	0.4189 (2)	0.22129 (17)	0.0440 (4)	
C14	0.7505 (2)	0.4552 (2)	0.32109 (18)	0.0487 (5)	
H14	0.8563	0.4704	0.2868	0.058*	
C15	0.8756 (3)	0.6431 (3)	0.4779 (2)	0.0728 (7)	
H15A	0.9786	0.6372	0.4438	0.087*	
H15B	0.8675	0.5876	0.5402	0.087*	
C16	0.8736 (4)	0.7952 (3)	0.5262 (2)	0.0831 (8)	
H16A	0.9657	0.8344	0.5850	0.125*	
H16B	0.7720	0.8003	0.5607	0.125*	
H16C	0.8819	0.8497	0.4642	0.125*	
C17	0.5999 (3)	0.3033 (3)	0.4376 (2)	0.0698 (6)	
H17A	0.5091	0.2600	0.3771	0.084*	
H17B	0.5723	0.3875	0.4843	0.084*	
C18	0.6251 (4)	0.1991 (4)	0.5128 (3)	0.1005 (10)	
H18A	0.5260	0.1717	0.5494	0.151*	
H18B	0.7155	0.2424	0.5721	0.151*	
H18C	0.6501	0.1153	0.4657	0.151*	
O1	0.57904 (18)	0.05021 (16)	0.17780 (15)	0.0657 (4)	
O2	0.74639 (16)	0.23833 (14)	0.12732 (12)	0.0502 (3)	
O3	0.74890 (16)	0.34407 (15)	0.38518 (12)	0.0535 (3)	
O4	0.73826 (17)	0.58602 (15)	0.38984 (13)	0.0578 (4)	
Br1	0.17952 (3)	0.54626 (3)	0.13035 (2)	0.07463 (13)	

### Atomic displacement parameters (Å^2^)

**Table:**

	*U*^11^	*U*^22^	*U*^33^	*U*^12^	*U*^13^	*U*^23^
C1	0.0560 (12)	0.0532 (12)	0.0665 (14)	0.0203 (10)	0.0098 (10)	0.0140 (10)
C2	0.0549 (13)	0.0715 (16)	0.0812 (16)	0.0235 (12)	0.0154 (12)	0.0195 (13)
C3	0.0660 (15)	0.0711 (16)	0.0759 (16)	0.0377 (13)	0.0041 (12)	0.0085 (13)
C4	0.0794 (17)	0.0487 (12)	0.0752 (15)	0.0271 (12)	0.0037 (12)	0.0142 (11)
C5	0.0601 (13)	0.0463 (12)	0.0606 (13)	0.0147 (10)	0.0032 (10)	0.0044 (10)
C6	0.0501 (11)	0.0403 (10)	0.0418 (10)	0.0145 (8)	0.0012 (8)	−0.0010 (8)
C7	0.0491 (11)	0.0401 (10)	0.0499 (11)	0.0116 (9)	0.0018 (9)	−0.0028 (9)
C8	0.0437 (10)	0.0399 (10)	0.0541 (12)	0.0137 (8)	0.0050 (8)	0.0073 (9)
C9	0.0616 (13)	0.0442 (11)	0.0495 (12)	0.0127 (10)	−0.0018 (10)	−0.0005 (9)
C10	0.0558 (12)	0.0502 (12)	0.0557 (12)	0.0102 (10)	−0.0116 (10)	0.0062 (10)
C11	0.0440 (10)	0.0445 (11)	0.0608 (13)	0.0146 (8)	−0.0032 (9)	0.0096 (9)
C12	0.0469 (10)	0.0429 (10)	0.0517 (11)	0.0150 (8)	−0.0022 (9)	0.0007 (9)
C13	0.0402 (9)	0.0417 (10)	0.0495 (11)	0.0105 (8)	−0.0001 (8)	0.0058 (8)
C14	0.0423 (10)	0.0471 (11)	0.0551 (12)	0.0118 (8)	−0.0024 (9)	0.0052 (9)
C15	0.0678 (15)	0.0647 (15)	0.0731 (16)	0.0076 (12)	−0.0277 (12)	−0.0026 (12)
C16	0.0846 (18)	0.0742 (17)	0.0724 (17)	0.0070 (14)	−0.0140 (14)	−0.0166 (13)
C17	0.0651 (15)	0.0801 (17)	0.0676 (15)	0.0161 (13)	0.0103 (12)	0.0209 (13)
C18	0.104 (2)	0.092 (2)	0.116 (2)	0.0156 (18)	0.0205 (19)	0.0523 (19)
O1	0.0468 (8)	0.0520 (9)	0.0981 (12)	0.0104 (7)	0.0066 (8)	0.0144 (8)
O2	0.0469 (7)	0.0433 (7)	0.0639 (9)	0.0181 (6)	0.0090 (6)	0.0086 (6)
O3	0.0459 (7)	0.0551 (8)	0.0607 (8)	0.0136 (6)	−0.0023 (6)	0.0135 (7)
O4	0.0532 (8)	0.0498 (8)	0.0622 (9)	0.0137 (6)	−0.0156 (7)	−0.0073 (7)
Br1	0.05575 (16)	0.07410 (19)	0.0983 (2)	0.03299 (12)	−0.00794 (12)	0.01173 (14)

### Geometric parameters (Å, º)

**Table:**

C1—C6	1.375 (3)	C11—Br1	1.8944 (19)
C1—C2	1.380 (3)	C12—C13	1.391 (3)
C1—H1	0.9300	C12—H12	0.9300
C2—C3	1.368 (3)	C13—C14	1.515 (3)
C2—H2	0.9300	C14—O4	1.397 (2)
C3—C4	1.366 (3)	C14—O3	1.405 (2)
C3—H3	0.9300	C14—H14	0.9800
C4—C5	1.382 (3)	C15—O4	1.432 (2)
C4—H4	0.9300	C15—C16	1.480 (3)
C5—C6	1.385 (3)	C15—H15A	0.9700
C5—H5	0.9300	C15—H15B	0.9700
C6—C7	1.480 (3)	C16—H16A	0.9600
C7—O1	1.193 (2)	C16—H16B	0.9600
C7—O2	1.364 (2)	C16—H16C	0.9600
C8—C9	1.374 (3)	C17—O3	1.427 (3)
C8—C13	1.384 (3)	C17—C18	1.475 (4)
C8—O2	1.402 (2)	C17—H17A	0.9700
C9—C10	1.377 (3)	C17—H17B	0.9700
C9—H9	0.9300	C18—H18A	0.9600
C10—C11	1.370 (3)	C18—H18B	0.9600
C10—H10	0.9300	C18—H18C	0.9600
C11—C12	1.374 (3)		
			
C6—C1—C2	120.4 (2)	C8—C13—C12	117.63 (17)
C6—C1—H1	119.8	C8—C13—C14	119.86 (16)
C2—C1—H1	119.8	C12—C13—C14	122.51 (17)
C3—C2—C1	120.1 (2)	O4—C14—O3	113.44 (17)
C3—C2—H2	120.0	O4—C14—C13	107.35 (15)
C1—C2—H2	120.0	O3—C14—C13	112.85 (15)
C4—C3—C2	120.0 (2)	O4—C14—H14	107.6
C4—C3—H3	120.0	O3—C14—H14	107.6
C2—C3—H3	120.0	C13—C14—H14	107.6
C3—C4—C5	120.6 (2)	O4—C15—C16	109.0 (2)
C3—C4—H4	119.7	O4—C15—H15A	109.9
C5—C4—H4	119.7	C16—C15—H15A	109.9
C4—C5—C6	119.5 (2)	O4—C15—H15B	109.9
C4—C5—H5	120.2	C16—C15—H15B	109.9
C6—C5—H5	120.2	H15A—C15—H15B	108.3
C1—C6—C5	119.40 (18)	C15—C16—H16A	109.5
C1—C6—C7	123.17 (18)	C15—C16—H16B	109.5
C5—C6—C7	117.43 (19)	H16A—C16—H16B	109.5
O1—C7—O2	122.72 (18)	C15—C16—H16C	109.5
O1—C7—C6	125.59 (19)	H16A—C16—H16C	109.5
O2—C7—C6	111.68 (17)	H16B—C16—H16C	109.5
C9—C8—C13	122.30 (17)	O3—C17—C18	108.7 (2)
C9—C8—O2	120.00 (17)	O3—C17—H17A	110.0
C13—C8—O2	117.64 (16)	C18—C17—H17A	110.0
C8—C9—C10	119.24 (18)	O3—C17—H17B	110.0
C8—C9—H9	120.4	C18—C17—H17B	110.0
C10—C9—H9	120.4	H17A—C17—H17B	108.3
C11—C10—C9	119.18 (18)	C17—C18—H18A	109.5
C11—C10—H10	120.4	C17—C18—H18B	109.5
C9—C10—H10	120.4	H18A—C18—H18B	109.5
C10—C11—C12	121.75 (18)	C17—C18—H18C	109.5
C10—C11—Br1	118.92 (14)	H18A—C18—H18C	109.5
C12—C11—Br1	119.33 (15)	H18B—C18—H18C	109.5
C11—C12—C13	119.83 (18)	C7—O2—C8	115.71 (15)
C11—C12—H12	120.1	C14—O3—C17	115.33 (16)
C13—C12—H12	120.1	C14—O4—C15	112.95 (16)

### Hydrogen-bond geometry (Å, º)

**Table:**

*D*—H···*A*	*D*—H	H···*A*	*D*···*A*	*D*—H···*A*
C1—H1···O2	0.93	2.42	2.740 (3)	100
C12—H12···O4	0.93	2.38	2.699 (2)	100
C17—H17*B*···O4	0.97	2.58	2.905 (3)	100
